# Novel TEVAR technique for thoracic aortic aneurysm repair: A case report

**DOI:** 10.1016/j.ijscr.2023.108651

**Published:** 2023-08-09

**Authors:** Jack Fritzke, Emmanuel Luciano, Akram Alashari, Merick Kirshner, Julio Rodriguez-Lopez

**Affiliations:** a Abrazo Arizona Heart Hospital, Phoenix, AZ, USA; b Biltmore Cardiology Abrazo Medical Group, Phoenix, AZ, USA; c Central Michigan University College of Medicine, Saginaw, MI, USA; d Department of Surgery, Central Michigan University College of Medicine, USA

**Keywords:** Case report, Thoracic aortic aneurysm, Thoracic endovascular repair, In-situ fenestration

## Abstract

**Introduction and importance:**

A thoracic aortic aneurysm (TAA) is a life-threatening condition affecting 5–10 per 100,000 people per year. If not repaired, mortality rates are reported as high as 11.8 %, increasing to 97 %–100 % following a TAA rupture. Thoracic endovascular aortic repairs (TEVAR) are becoming more common, but currently face limitations due to complex vasculature. New techniques may provide a safer alternative.

**Case presentation:**

70-year-old male presenting with a history of hypertension, dyslipidemia, and previous replacement of ascending aorta and hemi arch with reimplantation of innominate artery done in 2020. A CT scan done during routine interval monitoring of previous TAA repair demonstrated a new aneurysm, which was confirmed with CT angiogram. A novel TEVAR technique was used for repair. The patient tolerated this procedure well and was discharged from the ICU after six days.

**Clinical discussion:**

Open procedures and hybrid techniques for TAA repair are not always suitable for high-risk patients. Alternative parallel grafting techniques have shown promising early results but still lack clinical support and long-term data. Several small-scale studies and case reports have demonstrated the use of in-situ laser fenestrations in various settings, but none have demonstrated the ability to extend the landing zone as far as zone 0 for repair of a Type B TAA.

**Conclusion:**

The use of this novel technique may be considered suitable in high-risk patients with various subtypes of TAAs not suitable for open repair. More cases and clinical trials are needed to compare risks and long-term results to more commonly performed procedures.

## Highlights

• Novel TEVAR technique for type B thoracic aortic aneurysm repair
• Triple In-Situ Laser Fenestration
• Femoral to axillary cardiopulmonary bypass

## 1 Introduction

A thoracic aortic aneurysm (TAA) is a life-threatening condition in which there is abnormal dilation of a portion of the aorta due to wall weakness or secondary to dissection of the medial arterial layer. TAAs effect 5–10 per 100,000 people per year, with aneurysms of the descending aorta (Type B aneurysms) representing roughly 35 % of these cases [1]. Overall, yearly mortality rates have been reported as high as 11.8 % and increased to 97 %–100 % following a TAA rupture. To avoid fatalities TAAs are closely monitored and intervention is highly recommended when a rupture is likely [2]. When indicated, the current gold standard for TAA repair is total arch replacement (TAR), but newer options such as a hybrid approach and Thoracic Endovascular Aortic Repair (TEVAR) have been increasing in popularity [3]. The hybrid approach combines endovascular techniques with open repair to minimize the complexity of the procedure, especially when there is involvement of more distal areas of the aorta. Use of the TEVAR technique alone does not require opening the chest cavity and has been shown as a safe alternative to open surgery with better short term recovery outcomes than open procedures. However, its use is still limited by complex vasculature or inadequate landing zones [4]. This case report demonstrates novel use of TEVAR for a complicated type B TAA that included aortic arch vasculature. This work has been reported in line with the SCARE 2020 criteria [5].

## 2 Case report

**Fig. 1:**
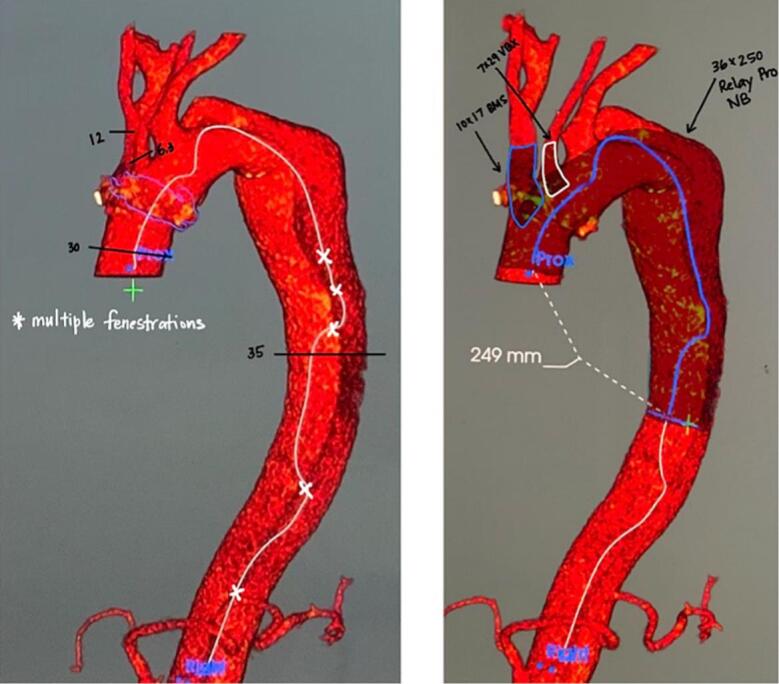
3D CT of thoracic aorta. * Indicates tear in medial layer of the vessel.

**Fig. 2:**
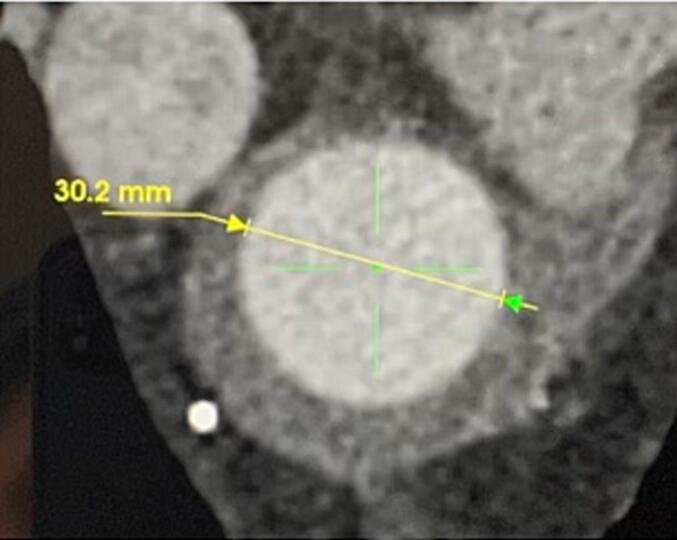
Proximal landing zone 1 cm proximal to the brachiocephalic trunk.

**Fig. 3:**
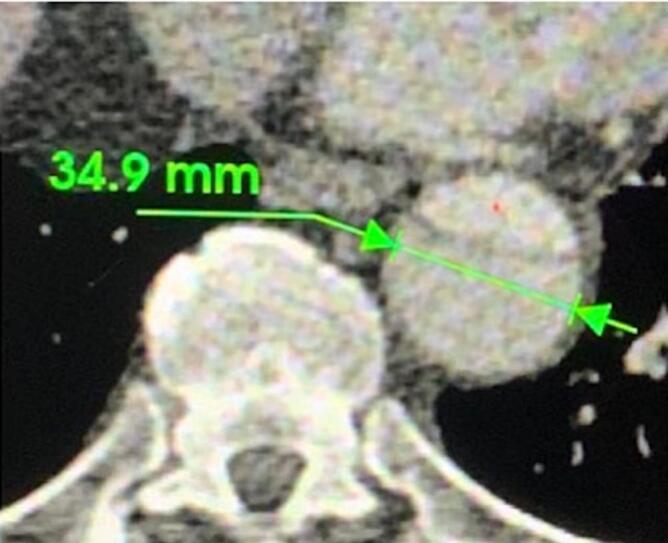
Distal landing zone diameter at proximal descending aorta.

**Fig. 4:**
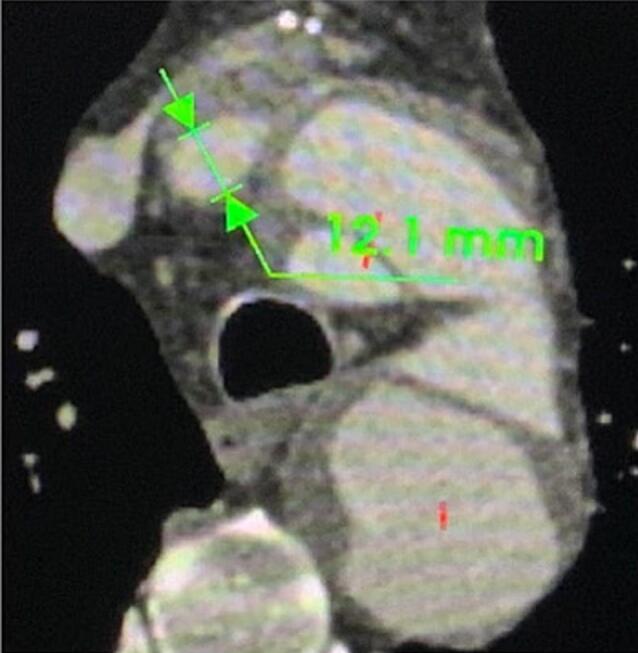
Brachiocephalic trunk diameter 12.1 mm.

**Fig. 5:**
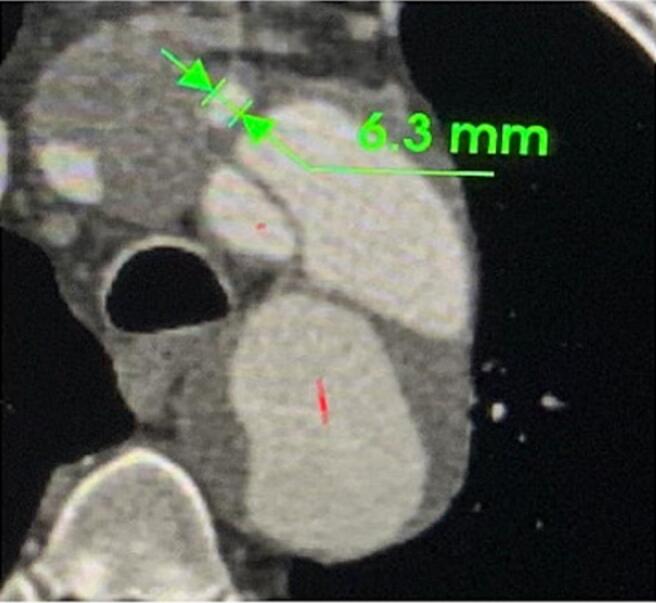
Left common carotid diameter 6.3 mm.

A 70-year-old male with a history of hypertension and dyslipidemia was being followed post replacement of the ascending aorta and hemi arch with reimplantation of the innominate artery two years ago. A recent CT scan for interval monitoring showed a false lumen of 5.1 cm at the proximal descending aortic arch with multiple fenestrations along the descending aorta, compared to a diameter of 4.2 cm a year prior. There was also evidence of a brachiocephalic artery pseudoaneurysm with an active channel that extended into the false lumen, further indicating the need for treatment. He denied any chest pain or shortness of breath at that time. Preemptive repair of the aneurysmal dilatation of the descending thoracic aorta was scheduled. Complications following the patient's previous arch repair made him a poor candidate for subsequent open repair. Lack of an adequate landing zone due to the aneurysm extending to the origin of the subclavian artery meant that the endograft would have to be placed in zone 0 (see Fig. 1, Fig. 2, Fig. 3, Fig. 4, Fig. 5).

**Diagram 1:**
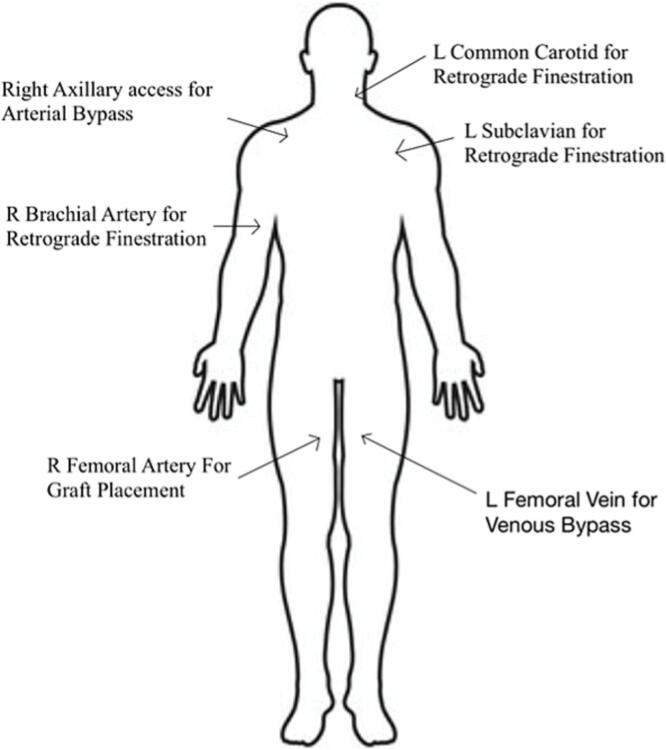
Venous and arterial access sites.

The operation was done by two separate teams. Once in the OR under adequate anesthesia, the right axillary artery and the left common carotid were exposed simultaneously via ultrasound guided technique by the two teams. The patient was prepped to go on cardiopulmonary bypass by anastomosis of an 8 French Hemashield graft to the axillary artery in end-to-side fashion using 5-0 Prolene suture for the arterial side and a 21 French Bio-Medicus cannula was placed in the left femoral vein for venous connection. Vascular surgery then gained access to the left subclavian and right brachial arteries. Ultrasound-guided access was performed on the right femoral artery, and an 18-gauge needle and 0.35 wire were used for cannulation and then upgraded to a Lunderquist wire. A 22 French dry seal Gore sheath was placed into the artery and advanced to the aorta. At this time, the patient was placed on partial femoral to right axillary bypass. Full heparinization was given (see Diagram 1).

**Fig. 6:**
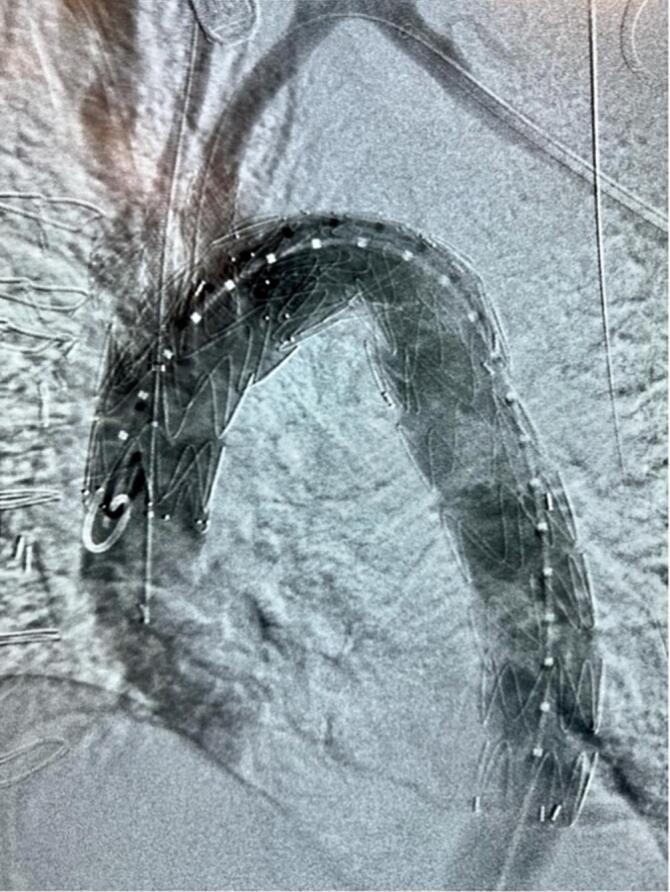
CT angiogram displaying blood flow following third stent placement.

Once bypass was initiated, a 23 mm turbo Elite laser was introduced through the 7 French sheath on the left common carotid artery and brought to the ostium of the vessel. Vascular surgery deployed a 34 × 150 mm Bolton relay graft across the arch ending at the proximal descending thoracic aorta. The laser was immediately adjusted for retrograde fenestration of the graft at 60/60 J of energy. A 7 mm balloon was used to dilate the opening. Angiogram confirmed flow from the aorta into the left Common Carotid Artery (CCA) before the VBX balloon expandable 7 × 39 mm covered stent was deployed within the fenestration 1 cm into the aortic lumen. The waist of this graft was cleared using a 10 × 40 mm balloon. Using the right axillary artery for access, similar technique was used for fenestration of the graft at the innominate artery. A 11 × 39 mm stent graft was placed and the ostium was flared using a 12 mm balloon. At this point a Bolton relay graft 36 mm × 200 mm was telescoped into the lumen of the previous graft and placed in the proximal to mid descending thoracic aorta. During this maneuver, balloon expansion of the innominate and CCA prevented deep formation of compression. Vascular surgery then returned to the subclavian artery. A 2.02 platelet laser was used to perform fenestration of the Bolton relay graft at the subclavian artery. This fenestration was predilated with a 9 mm balloon, an 11 × 39 mm stent graft was placed, and the centimeter sticking into the aorta side was flared using a 40 mm balloon (see Fig. 6).

Angiogram demonstrated excellent flow through all three aortic head vessels with no evidence of leak, dissection, or stenosis. The patient was weaned from cardiopulmonary bypass. Cannulas were removed and all openings were closed prior to the patient being taken to the ICU for close monitoring.

Recovery was uneventful aside from the development of a left neck hematoma which was resolved medically. The patient was discharged six days post-op on antiplatelet therapy with aspirin and instructed to follow-up with vascular surgery.

## 3 Clinical discussion

Affective use of TEVAR for Type B TAA requires a proximal landing zone of greater than 2 cm in order to minimize stent leaks or migration [6]. In this patient, the complex vascularity of the arches necessitated coverage of all three head vessels for an appropriate landing zone to be achieved near the aortic root. Several small-scale studies and case reports have demonstrated the usefulness of utilizing in-situ laser fenestrations during emergent procedures and in single arch vessel occlusion. However, none of these have demonstrated the ability to extend the landing zone as far as zone 0 for repair of a Type B TAA [7,8]. Parallel grafting techniques such as the chimney technique have shown promising early results for maintaining perfusion when covering vessels during TEVAR, but clinical support and long term data is still lacking for this to be a primary treatment option for complex TAAs [9].

The decision to put the patient on partial femoral to right axillary bypass was made to ensure cerebral perfusion during deployment of the primary aortic graft, reperfusion, and branch stenting. This helped to provide ample time for proper fenestration and stenting, as well as decreased risk if complications were to occur during this time. The total time spent on bypass during the procedure came to roughly 45 min. By obtaining vascular access to perform laser perforations, this technique also eliminates the need for a sternotomy or a thoracotomy to be performed. This makes the procedure suitable for patients who are unwilling or unable to undergo hybrid procedures in which a sternotomy is performed to debranch specific vasculature [10].

## 4 Conclusion

This procedure demonstrated that TEVAR with triple fenestrations of the left subclavian, left common, and innominate artery is a viable option for repair of a Type B aortic dissection with no adequate landing zone distal to zone 0. The use of this technique may also be suitable for ascending and aortic arch TAAs which require revascularization of the head vessels. To further elucidate the efficacy of retrograde laser fenestration for TAA repair, a larger sample size is needed. Due to the novelty of the procedure, it is currently not possible to determine if there is a lower risk of complications than open repair or parallel grafting techniques.

## Ethical approval

N/A, this was an isolated case report done with the consent of the patient. No further research studies are being pursued.

## Funding

N/A.

## Guarantor

Jack Fritzke.

## Registration of research studies

N/A.

## Declaration of competing interest

N/A.
